# How is trauma a transdiagnostic risk factor? A biopsychosocial model of risk and protective mechanisms following childhood trauma

**DOI:** 10.1192/j.eurpsy.2024.82

**Published:** 2024-08-27

**Authors:** H. Kerbage, D. Purper-Ouakil

**Affiliations:** ^1^University Hospital Center of Montpellier (CHU de MOntpellier), Montpellier; ^2^Center for Epidemiology and Population Health (CESP), Paris, France

## Abstract

**Abstract:**

Traumatic exposure is a common global problem across nations. It is currently well established that childhood trauma is associated with increased risk for psychopathology transdiagnostically, with children having experienced trauma being twice as likely to develop a mental health condition compared to those who have never experienced trauma. According to population-based studies, this heightened risk for the emergence of mental health disorders persists throughout adolescence and adulthood. The risk for psychopathology seems to be most marked in children exposed to interpersonal violence (child emotional and physical abuse, neglect, sexual violence). In this presentation, we will summarize the results of an increasing number of published studies that have examined the mechanisms underlying vulnerability to psychopathology following childhood trauma and protective factors that buffer this risk. Specifically, we will highlight the role of emotion dysregulation and interpersonal difficulties, related to disrupted threat processing following trauma exposure, in mediating the impact of trauma on internalizing and externalizing symptoms. Research studies have also identified protective factors accross the lifespan that might mitigate these outcomes, including social support and emotional skills building. Based on this review, we will suggest a conceptual transdiagnostic and biopsychosocial model of risk and resilience, which can provide opportunities and targets for early interventions and treatment, at the primary and secondary healthcare levels, as well as the social, public health and community levels. Our model is based on a socioecological and multisystemic paradigm of risk and resilience, where resilience is conceptualized as an interaction between individuals and resourceful environments and communities.

**Disclosure of Interest:**

None Declared

